# Captopril supported on magnetic graphene nitride, a sustainable and green catalyst for one-pot multicomponent synthesis of 2-amino-4*H*-chromene and 1,2,3,6-tetrahydropyrimidine

**DOI:** 10.1038/s41598-023-47794-2

**Published:** 2023-11-23

**Authors:** Fatemeh Rezaei, Heshmatollah Alinezhad, Behrooz Maleki

**Affiliations:** Department of Organic Chemistry, Faculty of Chemistry, University of Mazandaran, Babolsar, Iran

**Keywords:** Chemistry, Materials science, Nanoscience and technology

## Abstract

Captopril (CAP) is a safe, cost-effective, and environmentally organic compound that can be used as an effective organo-catalyst. Functional groups of captopril make it capable to attach to solid support and acting as promoters in organic transformations. In this work, captopril was attached to the surface of magnetic graphene nitride by employing a linker agent. The synthesized composite efficiently catalyzed two multicomponent reactions including the synthesis of 1,2,3,6-tetrahydropyrimidine and 2-amino-4*H*-chromene derivatives. A large library of functional targeted products was synthesized in mild reaction conditions. More importantly, this catalyst was stable and magnetically recycled and reused for at least five runs without losing catalytic activity.

## Introduction

In recent years, employing advanced sustainable catalytic system have attracted much attention as a green approach to overcome environmental pollution^1^. In this regard, bio-based catalytic systems have been introduced as promising compounds due to their nontoxicity, economy, biodegradability, and availability^2^. Through numerous research reports, the participation of small drugs and biomaterials, with multiple functional groups, in catalytic systems to perform organic reactions such as C–C and C-heteroatom bond forming reactions, cyclization, Knoevenagel condensation, and Michael addition have attracted much attention^3–6^.

One of the effective strategies to take advantage of these compounds and simultaneously benefit from the privilege of the heterogeneous catalytic system, including stability, reusability, and easy recovery, is the functionalization of solid supports with organic molecules and active biomolecules^7^. Carbon-based nanomaterials are known as interesting solid support compounds, specifically two-dimensional nanosheet constituents, and their unique structure has led to charming physicochemical properties^8^. Among them, graphitic carbon nitride (g-C_3_N_4_) is recognized as a layered polymer whose attractive properties such as high stability, eco-friendly property, large surface area, rich in N, and nontoxicity have converted it into a valuable and useful class of carbon nanomaterials^9,10^. Therefore, the widespread use of g-C_3_N_4_ sheets have been observed in various research fields consisting fuel cells, photoelectronic, water splitting, sensors, CO_2_ reduction, solar energy conversion, and heterogeneous catalysis^11–21^. However, applying functionalized g-C_3_N_4_ sheets in the catalytic field is an emerging topic; so, exploration of its new heterogeneous catalysts would be desirable and valuable. Several studies focused on the use of g-C_3_N_4_ for organic conversion, Mannich reaction, Knoevenagel condensation, reduction of N_2_ to ammonia, and degradation of organic contaminations^22–27^.

In particular, employing short and effective synthetic strategies can contribute to the development of green chemistry. Multicomponent reactions as a rapid synthesis strategy have concerned much attention as a green technique due to high atom economy, quick and easy performance, and saving time and energy^28^. Numerous research studies have indicated that g-C_3_N_4_ is a sufficient support material for the implementation of multicomponent reactions. For example, Daraie et al. reported the activity of a heterogenous catalyst of silver incorporated into g‑C_3_N_4_/Alginate in click coupling reactions^29^. In another research effort, 1,2,3,6-tetrazoloquinazolines were synthesized through an efficient regioselective multicomponent synthesis in the presence of sulfonated g-C_3_N_4_^30^. On the other hand, the tendency to reach an easy separatable and rapid recyclable catalyst encouraged scientist to use magnetic/g-C_3_N_4_. The nanocomposite of magnetic g-C_3_N_4_ was identified as an efficient catalyst for the one-pot multi-component synthesis of spirooxindoles^31^. In another research effort, a serious of multicomponent tandem reactions were carried out by employing sulfonated g-C_3_N_4_ as a solid bifunctional catalyst^32^. Moradi et al*.* reported an effective synthesis of 2-amino-4*H*-chromene derivatives catalyzed by magnetic/silica/g-C_3_N_4_^33^.

Currently, magnetic nanoparticles (MNPs) support obtaining a beneficial separation by magnetic force that magnetic separation process is more facile than some traditional separation methods, such as filtration or high-speed centrifugation^34,35^. However, to achieve more stable Fe_3_O_4_ NPs with diminished aggregation, surface modification was considered as an essential step in the catalyst preparation procedure. In addition, various natural, and synthesized polymer complexes coated with MNPs have been developed for high surface area, ease of preparation, more stable, userfriendly catalysts, and efficiency in a number of organic reactions^36–38^.

Captopril (CAP) is a biodegradable and nontoxic molecule with thiol and carboxy acid groups that can act as a green efficient catalyst. Captopril is one of L-proline derivatives, biodegradable, and nontoxic molecule with pharmacological properties. It is a low-cost, green, and easily accessible ligand with thiol, and carboxy acid groups that has been used as an acidic, and efficient catalyst.^39^ Herein, captopril was attached covalently on magnetic/g-C_3_N_4_, and after characterization, the efficiency of this stable and reusable catalyst was investigated in the synthesis of two series of valuable heterocyclic compounds via one-pot multicomponent reaction processes. 2-amino-4*H*-chromene derivatives are known as anti-fungal, anti-cancer, and anti-bacterial agents; moreover, extensive industrial usage in photoactive compounds, agricultural chemicals, and pigments has stimulated researchers to study their synthesis methods^40–42^. However, most of the published synthesis strategies suffer from some drawbacks, the need for high temperatures, long reaction times, and large amounts of catalyst^43–46^. In this regard and to overcome these problems, in the field of catalyst and multicomponent synthesis reactions approaches, several derivatives of 2-amino-tetrahydro-4*H*-chromene-3-carbonitriles were synthesized in the presence of magnetic/g-C_3_N_4_/Cap.

The catalytic activity of the introduced catalyst was also investigated in the synthesis of 1,2,3,6-tetrahydropyrimidines via a one-pot three-component condensation reaction. Rare reports on the preparation of these heterocyclic compounds suffer from the serious disadvantage of by-product formation^47–49^. To the best of our knowledge, successful efforts in the efficient synthesis of 1,2,3,6-tetrahydropyrimidines are limited to the use of a Zn-based catalytic system in this reaction^50^. Accordingly, this work is in the vanguard in the metal-free synthesis of these biologically active compounds^51^.

## Experimental

### General

All chemicals were purchased from Merck and Sigma Aldrich and used without purification. Electro thermal 9100 apparatus was used to determine of melting points. Sonication for synthesis of catalyst was performed by Elma at 60 Hz. The FT-IR Spectra were detected through Shimadzu IR-470 spectrophotometer. Raman spectroscopy was carried out using a Takram P50C0R10 Raman system. The ^1^H and ^13^C spectra of products were recorded with a Bruker DRX 400-Avance spectrometer. X-ray diffraction (XRD) pattern was recorded in Philips PW-1830. Magnetic analysis curves were attained by using VSM model MDKB from Danesh Pajohan Kavir Co. Kashan, Iran. The SEM images of the nanocatalyst were recorded via a MIRA_3_TESCAN-XMU instrument. TEM images were recorded with TEM Philips EM-208S, 100 kV. FEI TECNAI F20 instrument was applied for the achievement of high-resolution transmission electron microscopic (HRTEM) images. Elemental analysis of the nanocatalyst (EDS analysis) was done using TESCAN4992 instrument. Thermogravimetric analysis (TGA) was recorded by SDT Q600 V20.9 Build 20 instrument.

### ***Preparation of g-C***_***3***_***N***_***4***_*** nanosheets***

According to the reported method^2^. The melamine was heated at 550 °C in a furnace at a ramp of 2.5 °C min^−1^ in static air for 4 h so bulk g-C_3_N_4_ nanosheets powder was synthesized. Then bulk g-C_3_N_4_ (1.0 g) was treated in the mixture of 20.0 mL of HNO_3_ and 20.0 mL of H_2_SO_4_ at room temperature for 2 h. The mixture was diluted with 1.0 L of deionized H_2_O, and the obtained precipitate was filtered and washed several times with deionized water and dried at 60 °C. Then, treated bulk g-C_3_N_4_ (1.0 g) was dispersed in 100.0 mL of water/isopropanol (1:1) by sonication for approximately 6 h. Finally, to separate the residual unexfoliated g-C_3_N_4_ nanoparticles, the formed suspension was centrifuged (5000 rpm).

### ***Synthesis of Fe***_***3***_***O***_***4***_***@g-C***_***3***_***N***_***4***_

Magnetic graphite-like graphitic carbon nitride (g-C_3_N_4_) was prepared through known reported method^52^. Initially, graphitic carbon nitride (0.4 g) was dispersed in 50 ml deionized water (DI) for 4 h in ultrasonic conditions; then FeCl_3_·6H_2_O (4.68 g, 17.3 mmol) and FeCl_2_·4H_2_O (2.3 g, 18. 14 mmol) were added to the solution. Then, aqueous ammonia solution (15 ml, 25%) was added drop wise to the previous mixture until the pH reached 9–10. The mixture was stirred at 80 ℃ for 2 h under nitrogen atmosphere. The resulting black solid was separated by an external magnet and washed thoroughly with DI water and absolute ethanol, then dried at 50℃ for overnight to provide the Fe_3_O_4_@g-C_3_N_4_.

### ***Synthesis of Fe***_***3***_***O***_***4***_***@g-C***_***3***_***N***_***4***_***-PrBr***

The resulting Fe_3_O_4_@g-C_3_N_4_ (0.5 g) were dispersed in 15.0 mL of dry toluene in ultrasonic conditions then, 1,3-dibromopropane (10 mmol, 1.1 mL) and sodium iodide (0.5 mmol, 0.075 g) was added to the dispersed solution, and the reaction mixture was refluxed overnight under inert atmosphere. The resulted mixture was cooled to room temperature and obtained brown solid was collected by using an external magnet bar, washed with ethyl acetate, and dried at room temperature overnight.

### ***Synthesis of Fe***_***3***_***O***_***4***_***@g-C***_***3***_***N***_***4***_***-Pr-Cap***

Fe_3_O_4_@g-C_3_N_4_-PrBr (0.5 g) was added into a round bottom flask containing 15 mL of dry toluene and dispersed under ultrasonic conditions for 30 min. Then, sodium iodide (0.5 mmol, 0.075 g) and captopril (0.5 mmol, 0.11 g) were added into the mixture under reflux in toluene (110 °C) for 48 h, under inert atmosphere. The mixture was cooled to room temperature and the catalyst was separated magnetically and washed with absolute ethanol, and subsequently dried at room temperature for 12 h.

### General procedure for the one-pot synthesis of 2-amino-4H-chromene derivatives

Benzaldehydes (1.0 mmol), malononitrile or ethyl cyanoacetate (1.0 mmol), and *CH*- or *OH*-acids (1.0 mmol) were added in 3 mL of ethanol in presence of 20 mg of the catalyst. This mixture stirred for appropriate times at 50 °C. The progress of the reaction was monitored by TLC (ethyl acetate: n-hexane, 1:1). After completion of the reaction, the catalyst was separated using an external magnet bar, the reaction mixture was diluted with diethyl ether (15 mL) and dried with anhydrous MgSO_4_. Then the mixture was filtered and washed with diethyl ether and purified via recrystallization in ethanol to reach pure products.

### General procedure for the one-pot synthesis of 1,2,3,6-tetrahydropyrimidine

Benzaldehydes (1.2 mmol), EtOH (3 mL), and catalyst (20 mg) were added successively to a stirring mixture of diethyl acetylene dicarboxylate (1.0 mmol) and anilines (2.0 mmol). The mixture was stirred at 50 ℃ for appropriate times. The reaction progress was monitored by TLC. After completion of the reaction, the catalyst was separated, the reaction mixture was diluted with diethyl ether (15 mL) and dried with anhydrous MgSO_4_. Then the mixture was filtered and washed with diethyl ether and the product was purified through recrystallization in ethanol to reach pure products.

## Results and discussion

**Figure 1:**
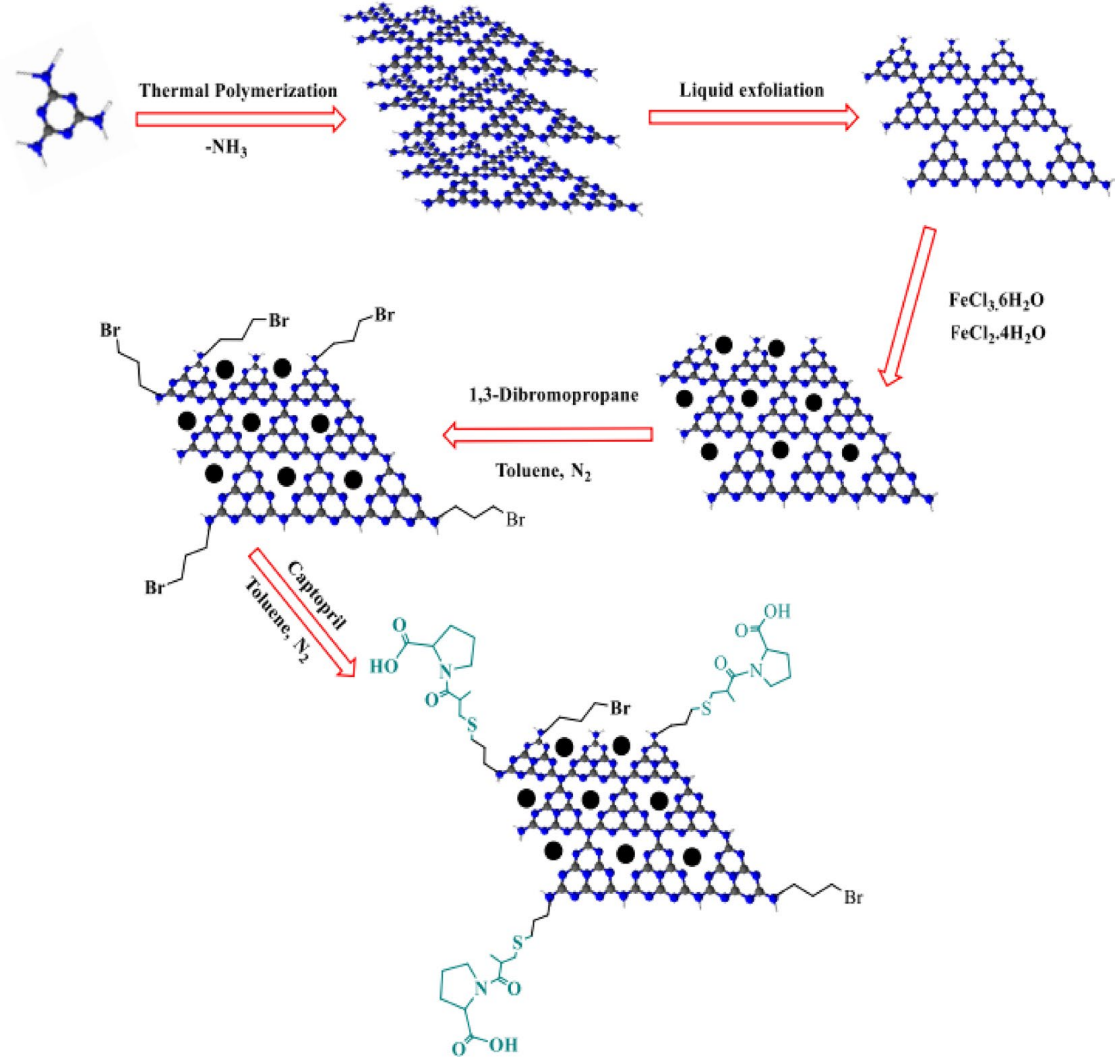
Preparation schematic of Fe_3_O_4_@g-C_3_N_4_-Pr-Cap.

The schematic of catalyst preparation has been illustrated in (Fig. 1) to prepare the Fe_3_O_4_@g-C_3_N_4_ nanoparticles, iron salts were added to g-C_3_N_4_ prepared from the thermal polymerization of melamine and liquid exfoliation process in flowing (Fig. 1). The presence of functional groups on the Fe_3_O_4_@g-C_3_N_4_, its surface was functionalized with 1,3-dibromopropane as a functionalizing agent to prepare Fe_3_O_4_@g-C_3_N_4_-PrBr. The immobilization of the captopril contains the carboxylic acid group on the magnetic graphitic carbon nitride for the first time is the main novelty of this work (Fe_3_O_4_@g-C_3_N_4_-Pr-Cap) as acidic nanocatalyst.

**Figure 2:**
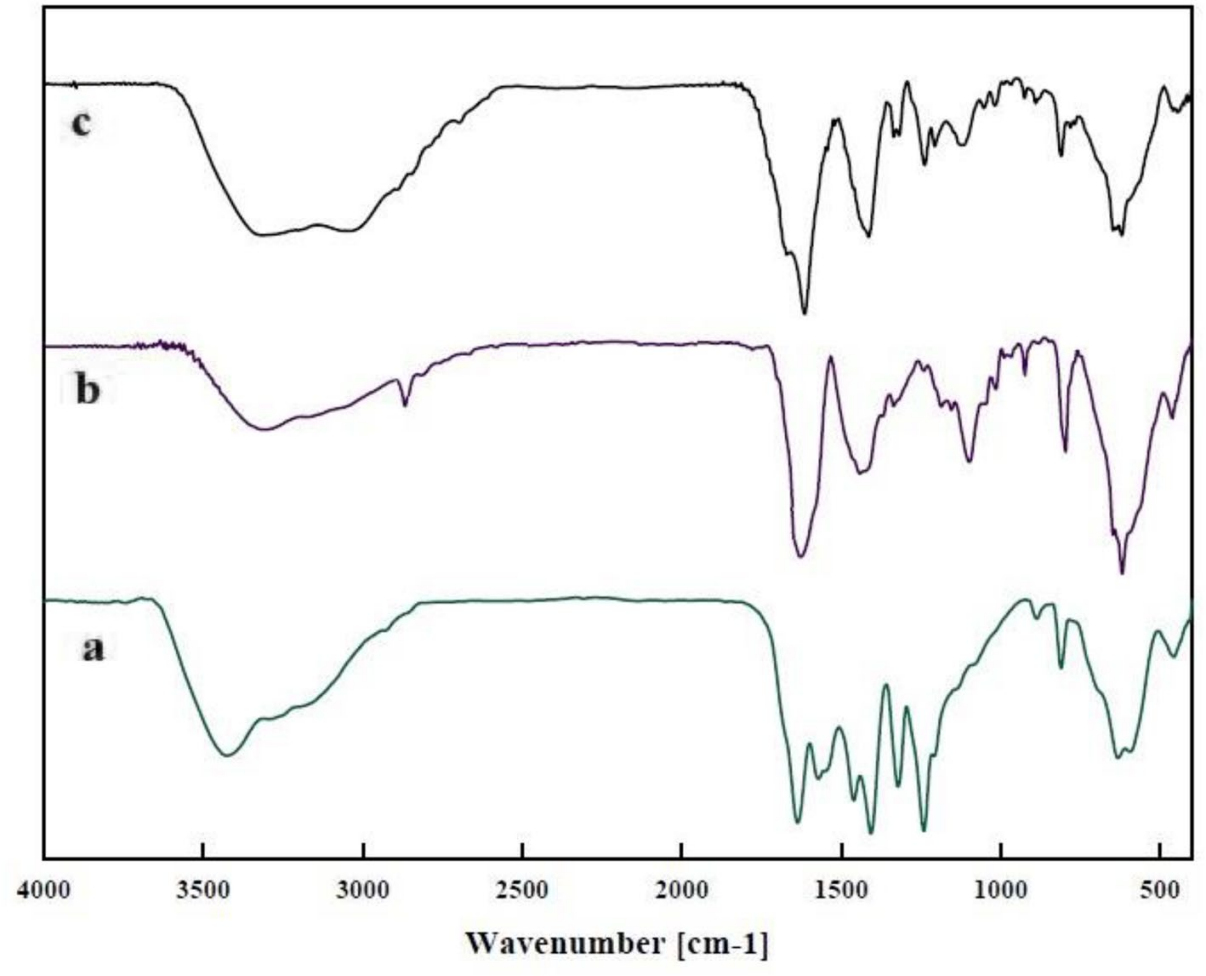
The FT-IR spectra of (**a**) Fe_3_O_4_@g-C_3_N_4_, (**b**) Fe_3_O_4_@g-C_3_N_4_-PrBr, (**c**) Fe_3_O_4_@g-C_3_N_4_-Pr-Cap.

The FT-IR spectroscopy was employed to characterize the synthesized particles and their modifications. In the spectrum of Fe_3_O_4_@g-C_3_N_4_ (Fig. 2a), a broad and strong peak of N–H group and O–H appeared around 2800 − 3600 cm^−1^, stretching vibration peaks of C=N were observed at 1637 and 1571 cm^−1^, stretching peaks of the C−N heterocycle were at around 1461, 1322, and 1241 cm^−1^, and a sharp peak at 810 cm^−1^ is due to the breathing vibration of tri-s-triazine units. The presence of a strong band at 565–632 cm^−1^ is related to the Fe–O band in the MNPs. The chemical structure of this intermediate compound, Fe_3_O_4_@g-C_3_N_4_-PrBr, was also confirmed by FT-IR (Fig. 2b). Observation of the main adsorption bands of g-C_3_N_4_ indicating the presence of basic g-C_3_N_4_ chemical structure. The spectrum of the final composite, Fe_3_O_4_@g-C_3_N_4_-Pr-Cap, is given in (Fig. 2c), the presence of 1690 cm^−1^ and 1307 cm^−1^ peaks of C=O and C–O bonds, respectively; approves the existence of captopril in the catalyst. The spectra of modified g-C_3_N_4_ also presented O–H and C–H bonds in the structure.

**Figure 3:**
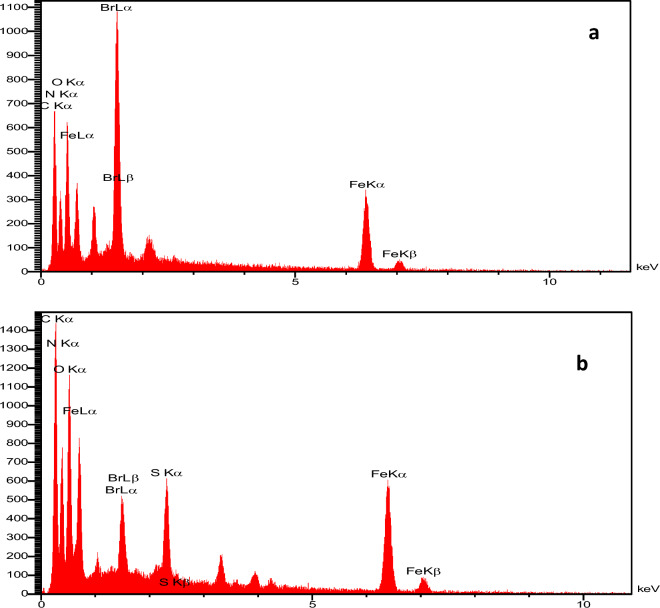
EDS spectra of Fe_3_O_4_@g-C_3_N_4_-PrBr (**a**), Fe_3_O_4_@g-C_3_N_4_-Pr-Cap (**b**).

The preparation of the intermediate compound, Fe_3_O_4_@g-C_3_N_4_-PrBr, and the final composite, Fe_3_O_4_@g-C_3_N_4_-Pr-Cap, was also approved by EDS. The EDS spectrum shows the composition of Br atoms in the framework (Fig. 3a) indicating linker attaches to magnetic graphitic carbon nitride. The EDS spectra of the final composite have also been depicted in (Fig. 3b), the existence of the constituent elements of this compound confirmed the formation.

**Figure 4:**
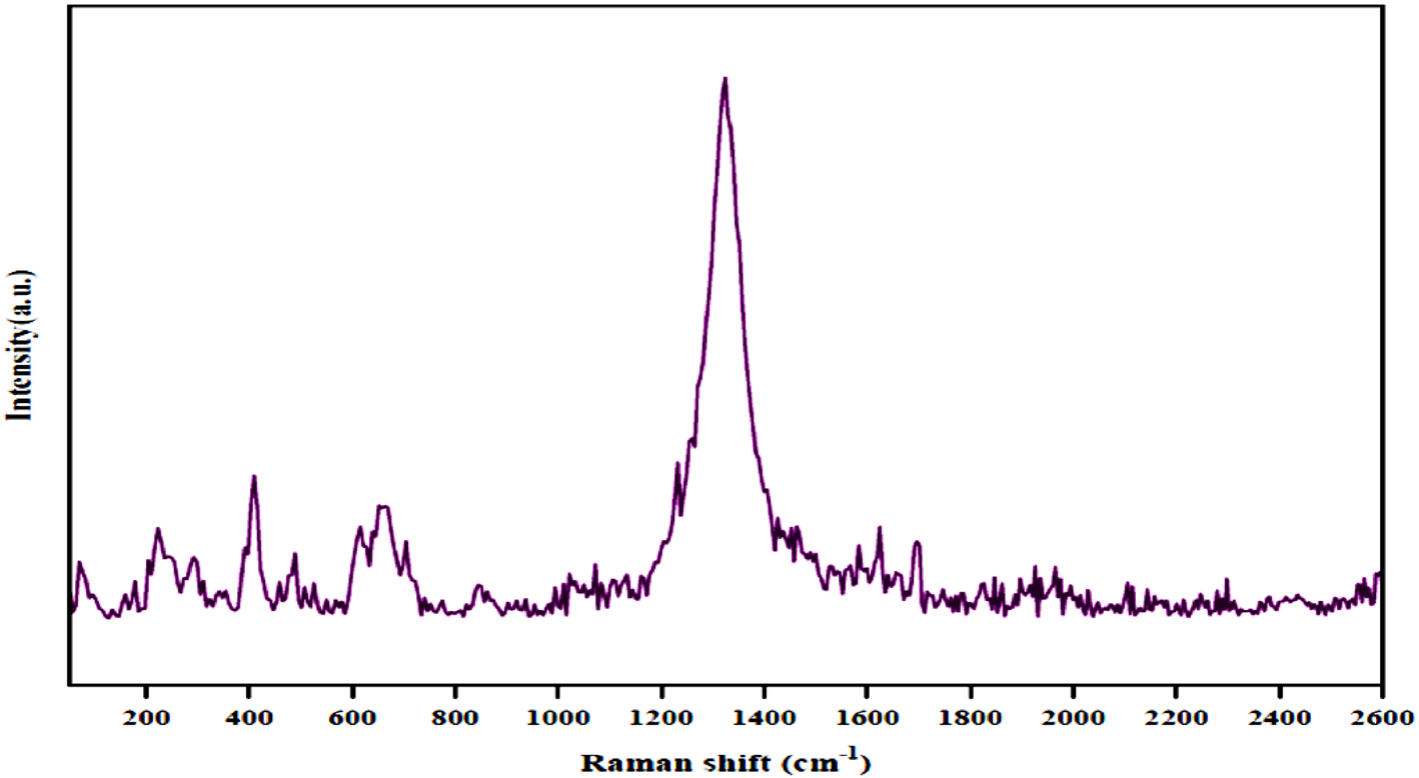
Raman spectra of Fe_3_O_4_@g-C_3_N_4_-Pr-Cap.

Raman was used to further illustrate the chemical structure of the Fe_3_O_4_@g-C_3_N_4_ nanocomposites (Fig. 4). Fe_3_O_4_@g-C_3_N_4_-Pr-Cap showed two main Raman bands at 223 and 652 cm^−1^ corresponding to vibrational modes of magnetite Fe3O4. In the Raman pattern of g-C_3_N_4_, 704 cm^−1^ was the typical 3-s-triazine ring breathing vibrational mode peak. Peak at 1325 cm^−1^ represented the D-band, and peak at 1583 cm^−1^ represented the G-band. Peak at 1624 and 1696 cm^−1^, indicating the presence of C=O groups of the captopril.

**Figure 5:**
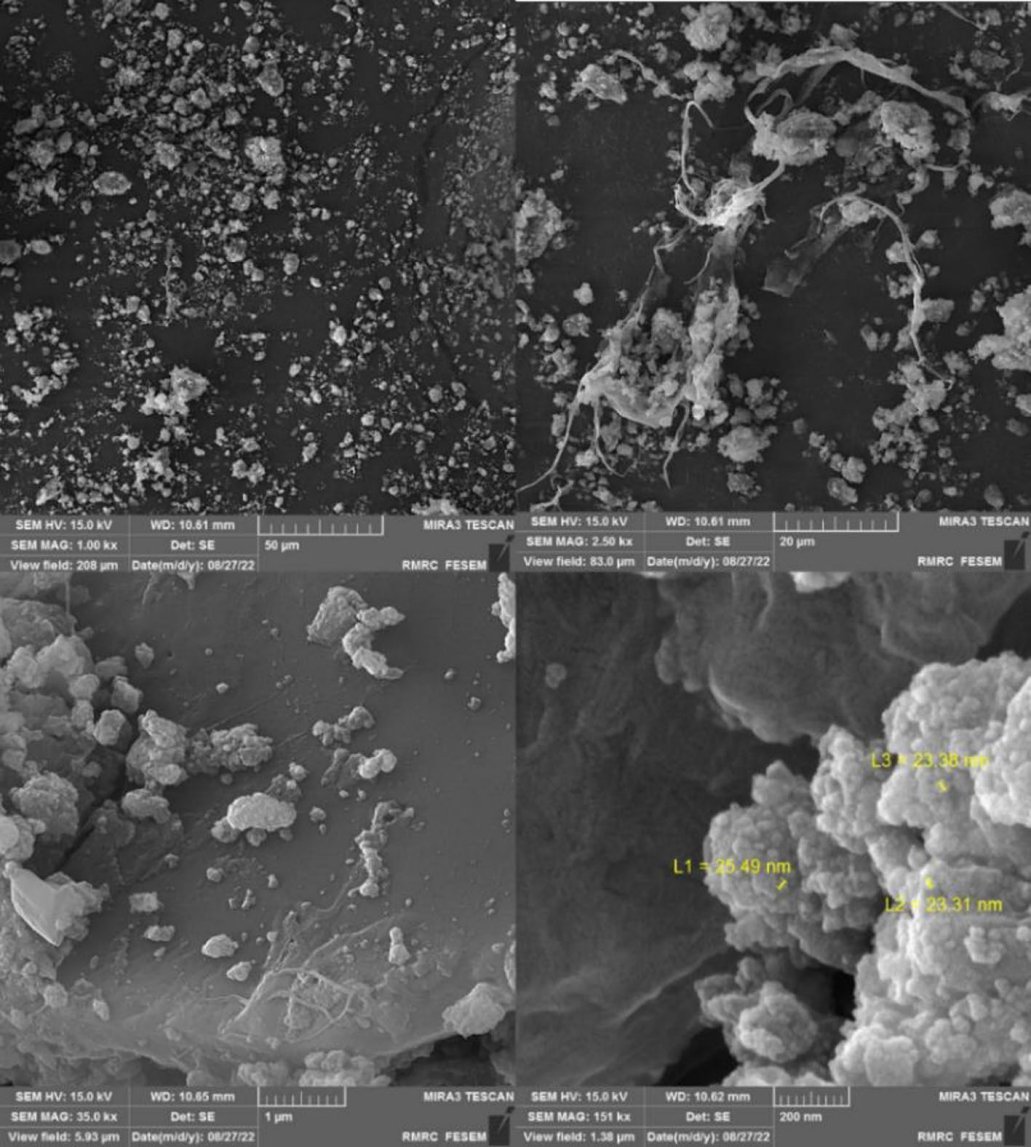
FE-SEM images of Fe_3_O_4_@g-C_3_N_4_-Pr-Cap.

**Figure 6:**
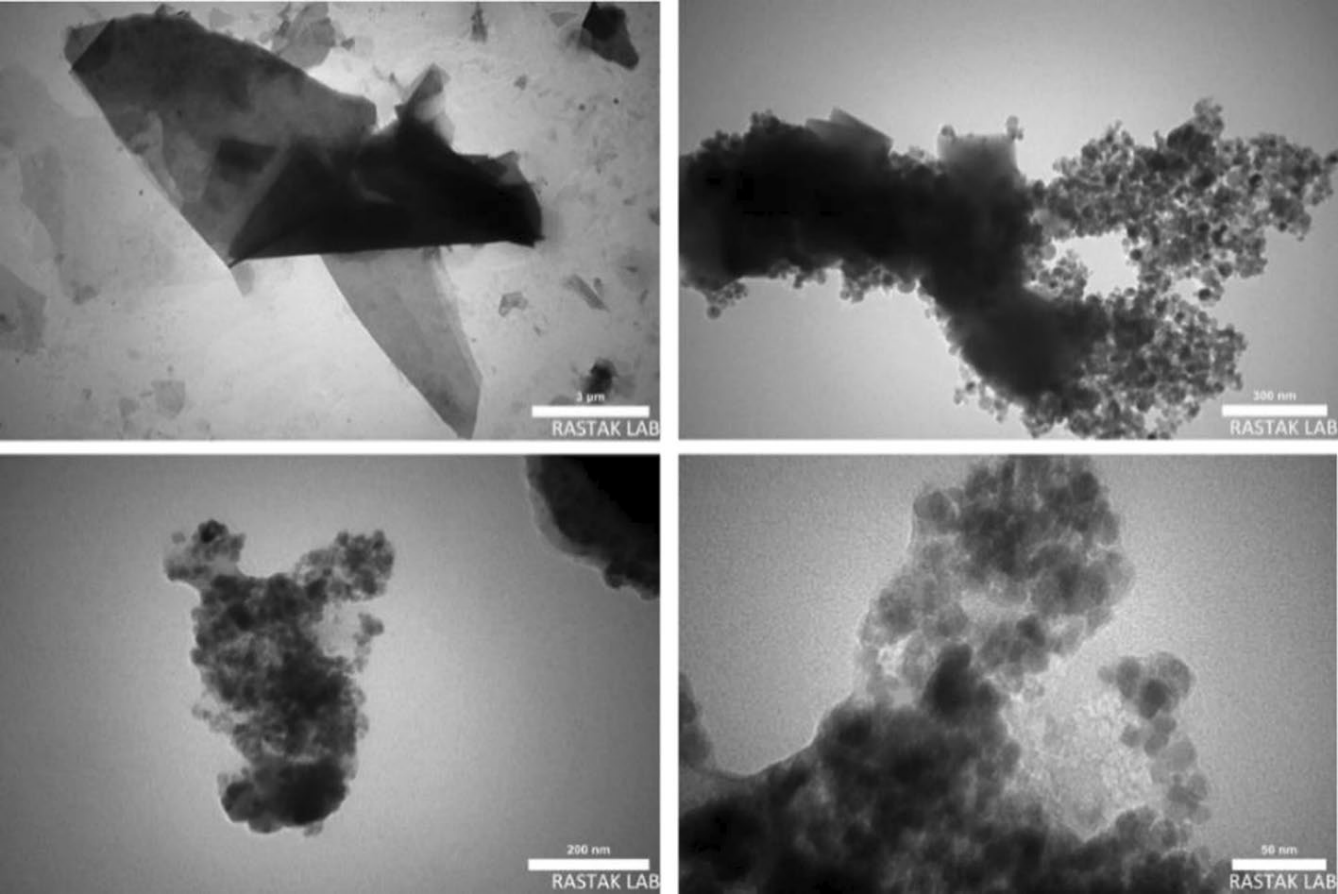
TEM images of Fe_3_O_4_@g-C_3_N_4_-Pr-Cap.

**Figure 7:**
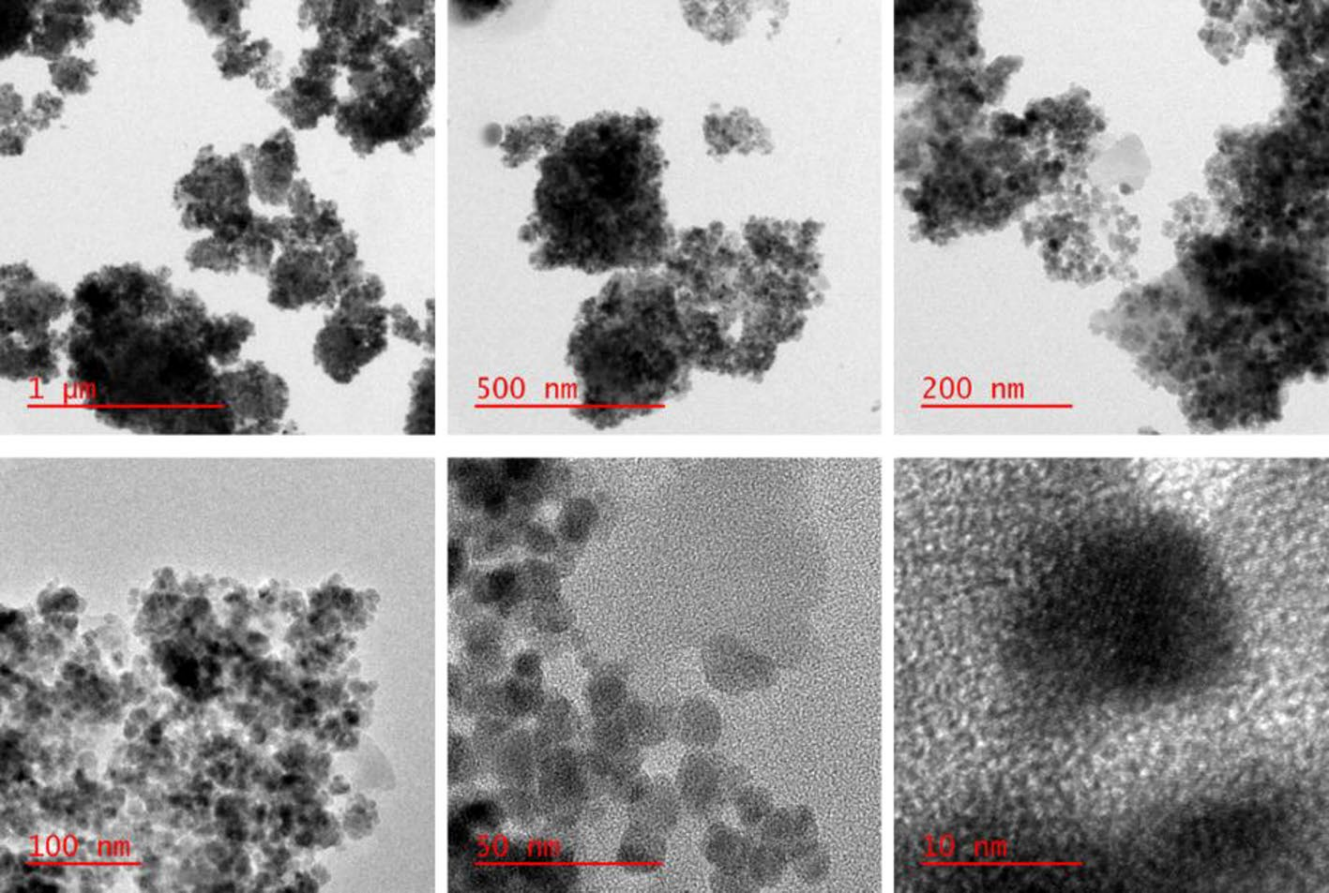
HR-TEM images of Fe_3_O_4_@g-C_3_N_4_-Pr-Cap.

FE-SEM, TEM, and HR-TEM analysis were applied to study the surface morphology of the catalyst, nano size, uniform distribution, and spherical shape of the particles have been illustrated in (Figs. 5, 6, and 7). Accordingly, the average diameter of nanoparticles was found less than 30 nm. The FE-SEM images obtained indicated wrinkled lamellar structure with relatively smooth surface, suggesting that the surface of Fe_3_O_4_@g-C_3_N_4_ sheets were immobilized with captopril.

**Figure 8:**
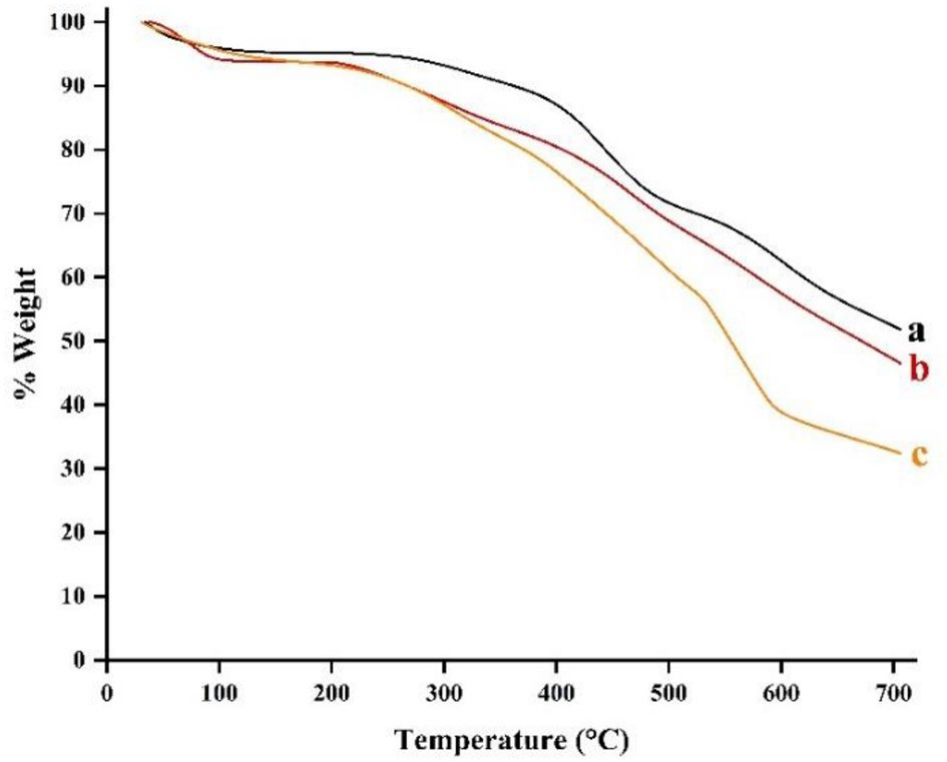
TGA plots of Fe_3_O_4_@g-C_3_N_4_ (**a**), Fe_3_O_4_@g-C_3_N_4_-PrBr-Cap (**b**), Fe_3_O_4_@g-C_3_N_4_-Pr-Cap (**c**).

The thermal behaviour of the prepared composite was investigated using thermogravimetric analysis, and decomposition cures of the final composite and its precursor have been depicted in (Fig. 8). The first weight loss step blew 200 $^\circ{\rm C}$, attributed to solvent and water releasing in all samples. The second step of weight loss in the range of 200–597 ℃ comes from the decomposition of organic groups of structures. The thermal stability of the synthesized composite was confirmed by low weight loss at higher temperatures.

The elemental analysis and the percent of weight loss in TGA results were used to calculate the amount of loaded organic species. Captopril is just a source of S in the synthesized composite; therefore, the amount of S (5.08 mmolg^−1^, from elemental analysis) was used to find captopril loaded amount (0.75 mmolg^−1^) in harmony with TGA results (0.78 mmolg^−1^).

**Figure 9:**
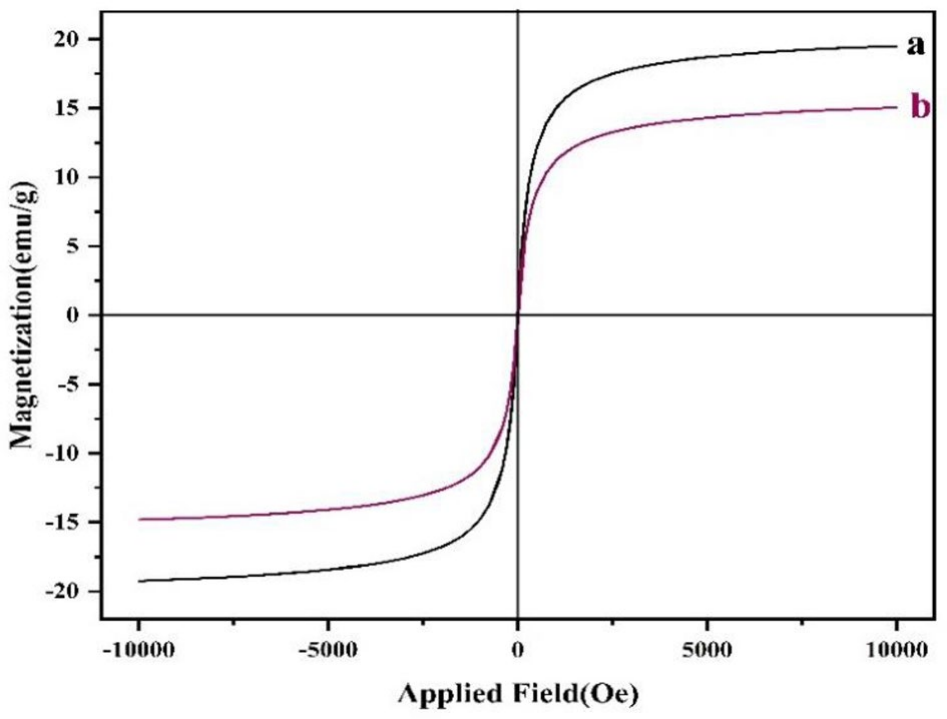
Magnetization curves of Fe_3_O_4_@g-C_3_N_4_ (**a**), Fe_3_O_4_@g-C_3_N_4_-Pr-Cap (**b**).

The magnetic properties of Fe_3_O_4_@g-C_3_N_4_ and the catalyst were investigated and the values were found as 19.25 emu g^−1^ and 14.89 emu g^−1^, receptively; their related curves are depicted in (Fig. 9). This paramagnetic activity of the catalyst was found to be lower than Fe_3_O_4_@g-C_3_N_4_ which may be related to the coating of magnetic graphene nitride with captopril.

**Figure 10:**
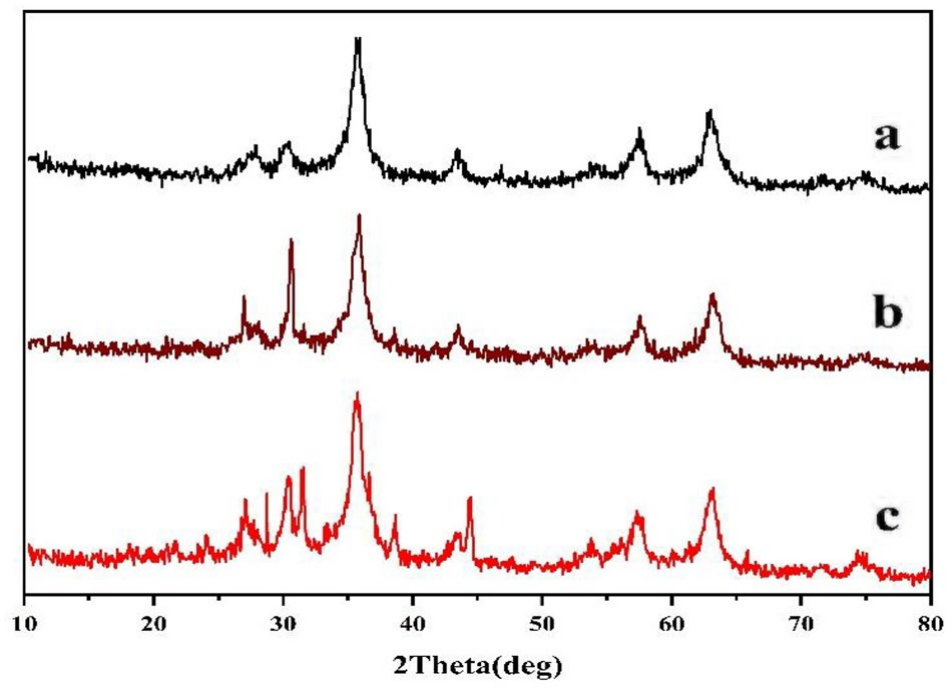
Fe_3_O_4_@g-C_3_N_4_ (**a**), Fe_3_O_4_@g-C_3_N_4_-PrBr-Cap (**b**), Fe_3_O_4_@g-C_3_N_4_-Pr-Cap (**c**).

The XRD spectrum of the catalyst and its precursor has been presented in (Fig. 10), distinguishing diffraction peaks at 2θ = 30.4°, 35.6°, 44.4°, 57.4°, 63.1°, and 74.6° are related to Fe_3_O_4_ nanoparticles with cubic phase. Moreover, the presence of conjugated aromatic systems with interplanar stacking crossponding to units g-C_3_N_4_ was depicted by existence of diffraction peaks at 2θ = 27.3°.

After synthesis and characterization of the final composite, its catalytic efficiency was investigated in MCR for 2-amino-4H‐chromenes synthesis. Initially, the MCR of benzaldehyde, malononitrile, and dimedone was selected as a model for reaction conditions optimization, including the study of the effluence of catalyst amount, the kind of solvent, and reaction temperature on reaction performance. The outcomes are listed in Table 1.

**Table 1:** Optimization for Synthesis of 2-amino-4*H*‐chromene^a^.

Entry	Solvent	Catalyst (mg)	t (min)	T (°C)	Yield^b^ (%)
1	EtOH	–	30	80	–
2	EtOH	Fe_3_O_4_@g-C_3_N_4_ (20)	30	80	28
3	EtOH	Fe_3_O_4_@ g-C_3_N_4_-PrBr (20)	30	80	32
4	EtOH	Fe_3_O_4_ (20)	30	80	Trace
5	EtOH	Captopril (20)	30	80	97
6	EtOH	Fe_3_O_4_@g-C_3_N_4_-Pr-Cap (20)	30	80	95
7	EtOH	Fe_3_O_4_@g-C_3_N_4_-Pr-Cap (30)	30	80	95
8	EtOH	Fe_3_O_4_@g-C_3_N_4_-Pr-Cap (10)	30	80	83
9	MeOH	Fe_3_O_4_@g-C_3_N_4_-Pr-Cap (20)	30	80	86
10	DMF	Fe_3_O_4_@g-C_3_N_4_-Pr-Cap (20)	30	80	73
11	H_2_O	Fe_3_O_4_@g-C_3_N_4_-Pr-Cap (20)	30	80	80
12	Solvent free	Fe_3_O_4_@g-C_3_N_4_-Pr-Cap (20)	30	80	44
13	EtOH	Fe_3_O_4_@g-C_3_N_4_-Pr-Cap (20)	30	50	95
14	EtOH	Fe_3_O_4_@g-C_3_N_4_-Pr-Cap (20)	30	25	82
15	EtOH	Fe_3_O_4_@g-C_3_N_4_-Pr-Cap (20)	15	50	95

^a^Reaction conditions: benzaldehyde (1.0 mmol), malononitrile (1.0 mmol), dimedone (1.0 mmol), and solvent (3.0 ml).

^b^Isolated yield.

For screening of the catalysts, the reaction was performed in ethanol at 80 °C, without any catalyst, and in the presence of pure magnetic nanoparticles, the reaction was intact (Table 1, entries 1, 4). Employing Fe_3_O_4_@g-C_3_N_4_ and Fe_3_O_4_@g-C_3_N_4_-PrBr afforded negligible conversion (Table 1, entries 2, 3). Pure captopril, safe, easily accessible, and low-cost organic compound, exhibited significant efficiency in model reaction (Table 1, entry 5) approved its property; however, considering green chemistry supporting from easy recyclable bed to generation of heterogenous catalyst is a more desirable approach. Interestingly, the magnetic nanoparticle-supported captopril was shown excellent activity in the intended reaction (Table 1, entry 6). After screening the catalyst, the effect of catalyst amount was investigated, more amount of catalysts didn’t give better results (Table 1, entry 7), and lower amounts of catalyst gave weak performance (Table 1, entry 8).

Through the advance of green chemistry, using green media in chemical reactions has been converted into an attractive target in scientific efforts. In this regard, in comparison with flammable and volatile organic solvents, DMF, MeOH, and EtOH are considered safe solvents (Table 1, entries 9, 10). However, among them, EtOH was recognized as a more economical, ecological, and versatile solvent that created suitable reaction conditions to achieve excellent performance, but the application of water and solvent-free media was not succeeded (Table 1, entries 11, 12). Low-temperature reactions are considered a green strategy due to cost-effectiveness, less energy consumption, and less chance of byproduct formation. The reaction was performed in milder conditions (Table 1, entry 13). Paying attention to the reaction time reached out to this conclusion that the reaction was completed in less time (Table 1, entry 15).

Optimization reaction conditions in hands, the generality of the catalyst was investigated through the synthesis of several derivatives. The chemical structure of processors, products, and reaction results have been presented in Table 2. Employing several derivatives of benzaldehyde, other components of the reaction keeping the same (Table 2, entries 1–12), with different electronic natures indicating that their electronic properties are not very important points in this reaction, resulting in high reaction yields and short reaction time for all cases. However, replacing dimedone with other diketones led to longer reaction times (Table 2, entries 13–19). As shown in Table 2, in all cases, the products were formed in high to excellent yields (89–97%). In general, our sustainable catalytic system was found as a very active and efficient catalyst for the synthesis of a series of heterocyclic compounds. A gram scale 2‐amino‐4*H*‐chromene reaction was carried out using optimized reaction conditions. The 2‐amino‐4*H*‐chromene reaction (Table 2, entrie 5) of benzaldehyde (1.6 g, 15 mmol), malononitrile (1.0 g, 15 mmol), and dimedone (2.1 g, 15 mmol), in presence of EtOH (45 mL) using 0.3 g of the catalyst at 50 °C was conducted. When the scale of the reaction was increased to 15 mmol, the reaction was still found to proceed successfully and the corresponding product was obtained in 90% yield.

**Table 2:** Synthesis of 2-amino-4*H*‐chromene derivatives using the catalyst^a^.


Entry	R	X	*CH*- or *OH*- acids 3	Products	Time (min)	Yield^b^ (%)	Mp (°C)	
							Observed	Literature
1	4-CN-C_6_H_4_	CN	Dimedone	**4a**	3	94	228–230	229–231^53^
2	4-NO_2_-C_6_H_4_	CN	Dimedone	**4b**	2	97	175–178	176–183^54^
3	4-CHO-C_6_H_4_	CN	Dimedone	**4c**	5	96	271–273	273–275^55^
4	4-Cl-C_6_H_4_	CN	Dimedone	**4d**	3	97	212–215	214–216^54^
5	C_6_H_5_	CN	Dimedone	**4e**	4	95	258–261	257–259^54^
6	4-OH-C_6_H_4_	CN	Dimedone	**4f**	4	92	224–227	224–226^53^
7	4-NMe_2_-C_6_H_4_	CN	Dimedone	**4g**	4	93	211–213	209–210^54^
8	Furan-2-yl	CN	Dimedone	**4h**	10	91	224–226	221–222^56^
9	Thiophene-2-yl	CN	Dimedone	**4i**	10	86	213–215	210–212^57^
10	4-CN-C_6_H_4_	CO_2_Et	Dimedone	**4j**	4	92	179–182	181–183^56^
11	4-NO_2_-C_6_H_4_	CO_2_Et	Dimedone	**4k**	5	91	152–154	153–154^58^
12	4-Cl-C_6_H_4_	CO_2_Et	Dimedone	**4l**	5	94	153–155	153–156^58^
13	C_6_H_5_	CO_2_Et	Dimedone	**4m**	7	93	149–152	148–150^58^
14	4-NMe_2_-C_6_H_4_	CO_2_Et	Dimedone	**4n**	7	93	155–157	156–157^59^
15	4-Cl-C_6_H_4_	CN	Ethyl acetoacetate	**4o**	12	90	171–173	172–174^60^
16	4-Cl-C_6_H_4_	CN	6-Methyl-2H-pyran-2,4(3H)-dione	**4p**	15	95	230–234	230–232^53^
17	4-Cl-C_6_H_4_	CN	Barbituric acid	**4q**	12	94	233–236	234–236^53^
18	4-Cl-C_6_H_4_	CN	3-Methyl-4H-pyrazole-5(4H)-one	**4r**	15	89	232–234	234–236^60^
19	4-Cl-C_6_H_4_	CN	1,3-Indandione	**4s**	15	97	288–291	287–289^61^
20	4-Cl-C_6_H_4_	CN	4-Hydroxycoumarin	**4t**	10	95	264–266	263–265^53^
21	4-Cl-C_6_H_4_	CN	2-Naphthol	**4v**	15	93	206–209	205–206^62^
22	Furan-2-yl	CN	2-Naphthol	**4u**	15	88	220–223	220–222^56^
23	Thiophene-2-yl	CN	2-Naphthol	**4w**	15	85	260–260	258–259^56^

^a^Reaction carried out with benzaldehydes (1.0 mmol), malononitrile or ethyl cyanoacetate (1.0 mmol) and Diketone (1.0 mmol**)** in a 3.0 mL EtOH using 20 mg of the catalyst at 50 °C.

^b^Isolated yield.

^c^Reaction carried out with terephthalaldehyde (1.0 mmol), malononitrile (2.0 mmol) and dimedone (2.0 mmol), in 3.0 mL EtOH using 20 mg of the catalyst at 50 °C.

**Figure 11:**
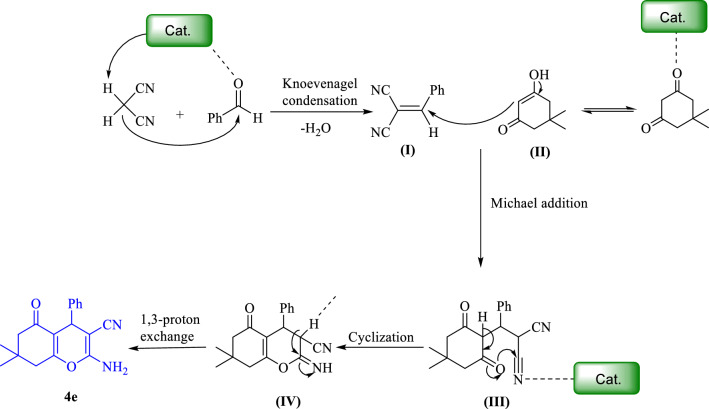
Proposed mechanism 2‐amino‐4*H*‐chromenes synthesis in the presence of the catalyst.

The proposed mechanism of the 2-amino-4*H*‐chromene synthesis in the presence of Fe_3_O_4_@g-C_3_N_4_-Pr-Cap has been depicted in (Fig. 11). Captopril supported on magnetic graphene nitride has several acidic catalytic active sites which activate benzaldehyde. Then, malononitrile reacted with the active carbonyl of benzaldehyde, Knoevenagel condensation, Michael addition, and final cyclization leading to the final product.

As a comparison study, the activity of Fe_3_O_4_@g-C_3_N_4_-Pr-Cap has been compared with reported catalytic systems applied for the one-pot multicomponent reaction for 2‐amino‐4*H*‐chromenes synthesis. The results are listed in Table 3, as can be seen, our catalytic system illustrated high efficiency at extremely short reaction time in desirable mild reaction conditions.

**Table 3:** Comparison of our catalyst with previous reported catalysts^a^.

Entry	Catalyst (mg)	Solvent	Time (min)	Yield %^a^	References
1	Melamine	H_2_O/EtOH	25	93	^63^
2	Aminopropylated silica gel	H_2_O	90	89	^64^
3	CuFe_2_O_4_@strach	EtOH	20	96	^65^
4	Fe_3_O_4_@MCM‐41@Zr‐piperazine	H_2_O/EtOH	30	85	^66^
5	2-Aminopyrine	EtOH	8	92	^67^
6	MNPs-GO-CysA	H_2_O/EtOH	15	93	^68^
7	Fe_3_O_4_@SiO_2_-creatine	EtOH	6	94	^43^
8	Fe_3_O_4_@g-C_3_N_4_-Pr-Cap	EtOH	3	96	This work

^a^All reactions were performed employing 4-chlorobenzaldehyde (1.0 mmol), malononitrile (1.1 mmol) and dimedone (1.0 mmol).

Encouraged by excellent outcomes of 2‐amino‐4H‐chromenes synthesis investigation and optimization reaction conditions in hands (Cat. 20 mg, EtOH (3 mL), 50 °C) of the 1,2,3,6-tetrahydropyrimidine derivatives synthesis was performed. Benzaldehydes with diverse electronic properties substituents reacted competently with anilines and diethyl acetylenedicarboxylate, and high yields of products were attained (Table 4). Accordingly, starting materials with different electronic natures did not exhibited any important effect on the reaction yields.

**Table 4:** Synthesis of 1,2,3,6-tetrahydropyrimidine derivatives using the catalyst^a^.


Entry	R	Products	Time (h)	Yield^b^ (%)	Mp (°C)	
					Observed	Literature
1	NO_2_	**7a**	8	86	169–171	–
2	F	**7b**	8	79	175–178	176–183^50^
3	Br	**7c**	8	69	162–165	164–166^50^
4	H	**7d**	8	80	167–169	166–169^50^
5	Me	**7e**	8	83	151–154	150–153^50^

^a^Reaction carried out with anilines (2.0 mmol), diethyl acetylenedicarboxylate (1.0 mmol) and benzaldehydes (1.2 mmol), in a 3.0 mL EtOH using 20 mg of the catalyst at 50 °C.

^b^Isolated yield.

**Figure 12:**
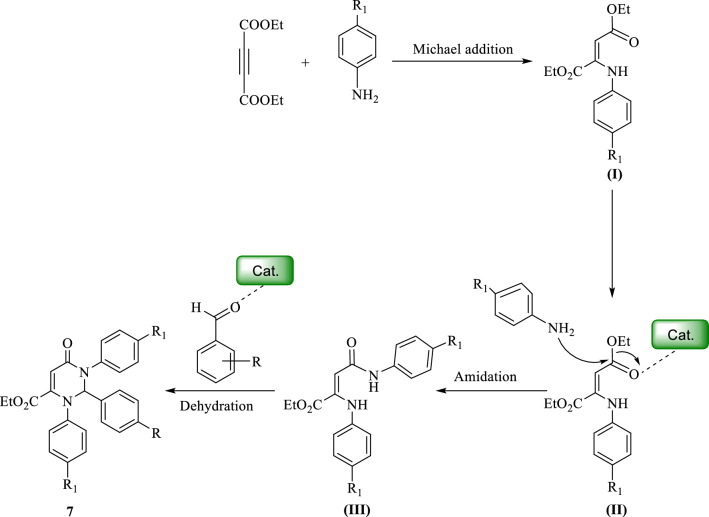
Proposed mechanism 2‐amino‐4H‐chromenes synthesis in the presence of the catalyst.

A plausible mechanism of 1,2,3,6-tetrahydropyrimidine synthesis has been given in (Fig. 12). Hydroamination occurred in the first step and amidation was performed in the next step by acid activation assistance. The product was formed in the last step through aldehyde dehydration and cyclization processes.

**Figure 13:**
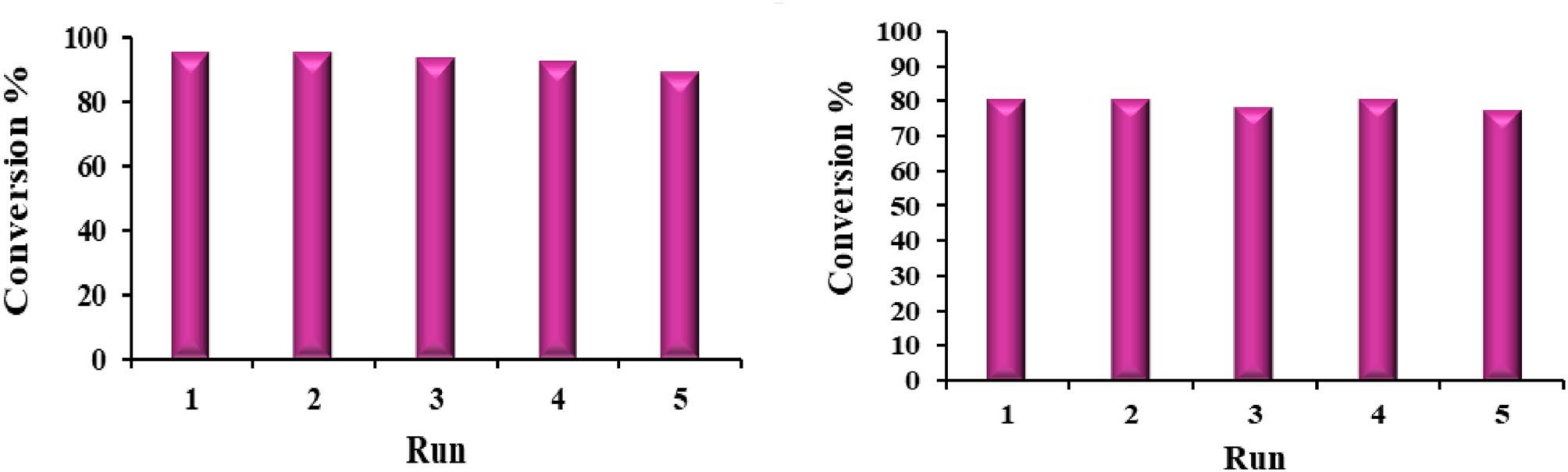
Recyclability of the catalyst in 2-amino-4*H*‐chromene (left) and 1,2,3,6- tetrahydropyrimidine (right) synthesis model reaction.

**Figure 14:**
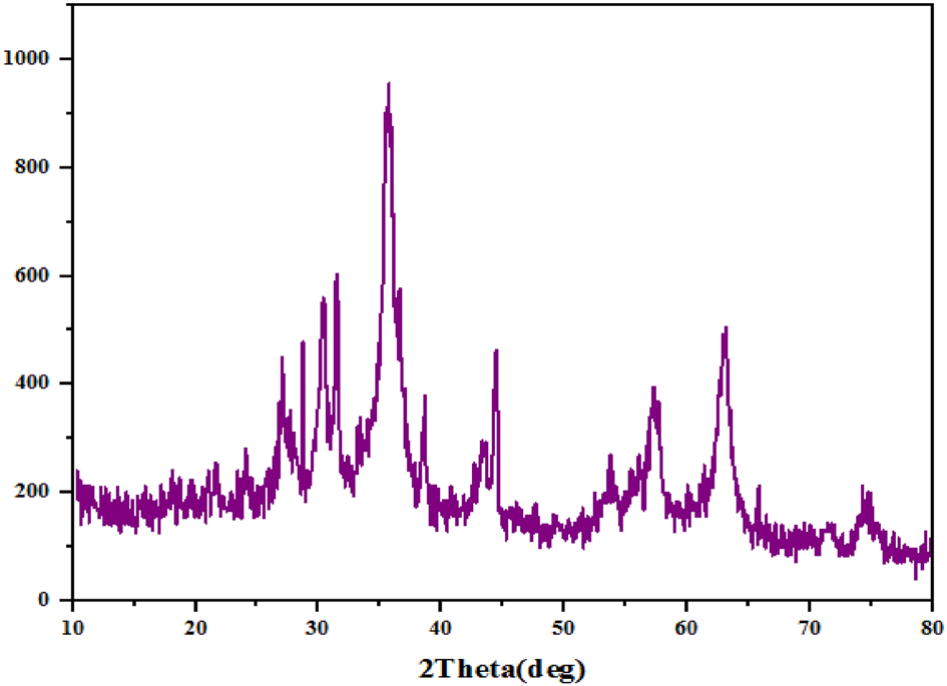
XRD of recycled catalyst after 5 runs.

**Figure 15:**
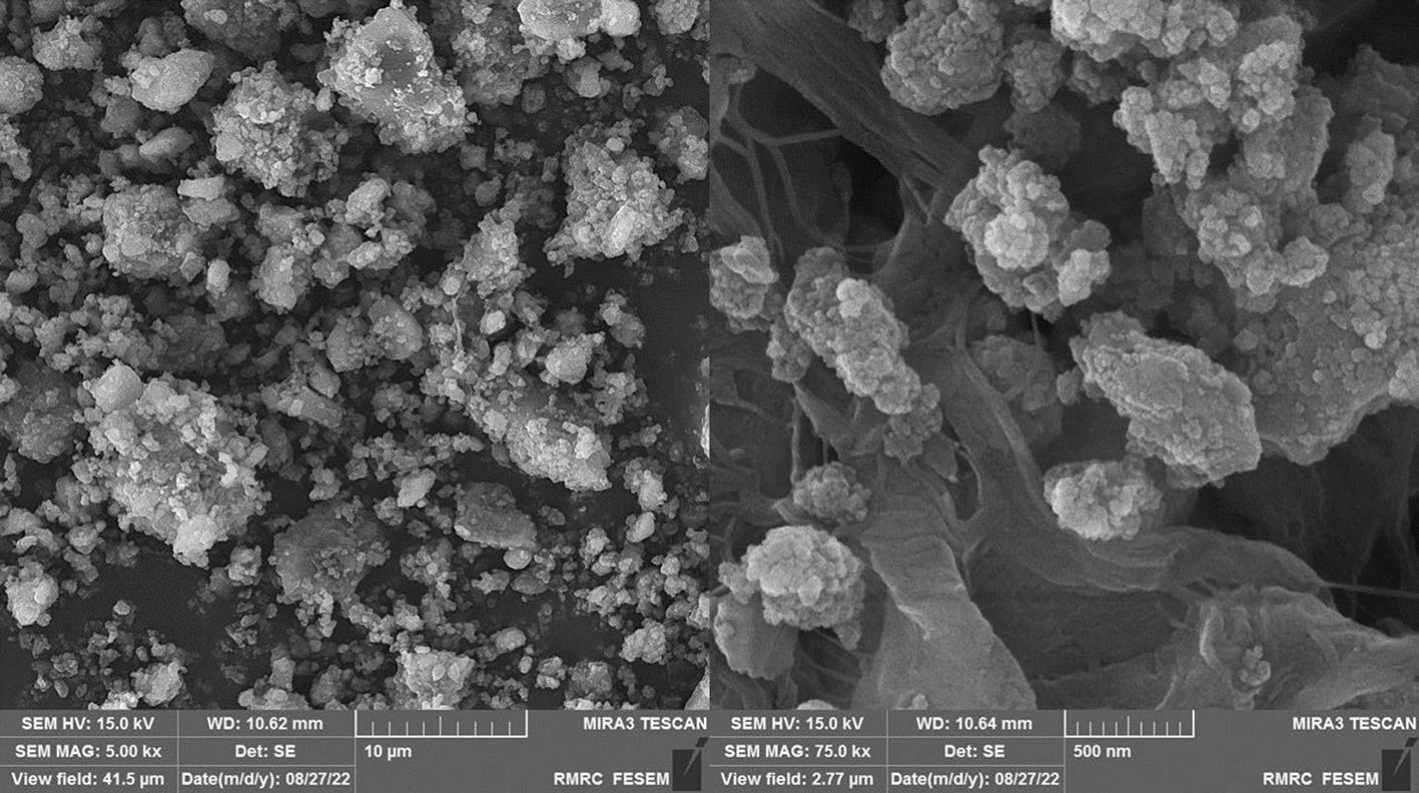
SEM images of recycled catalyst.

Difficult separation of homogeneous catalysts is known as a serious problem in chemical transportation due to their economic and environmental disadvantages. In this work, a heterogenous magnetic catalyst was introduced, and after an investigation of its efficiency, its recyclability was studied. For this purpose, after the reaction completion, separation of the catalyst from the reaction mixture was done by employing an external magnet bar, washed, and reused in another fresh reaction mixture. The performance of reused catalysts for both studied reactions has been reported in (Fig. 13). The catalyst exhibited significant activity even after five times using. The recycled catalyst was characterized through several analysis methods including XRD, and SEM. The results are reported in (Figs. 14 and 15), these results indicated that no significant changes were observed in comparison with fresh catalyst.

**Figure 16:**
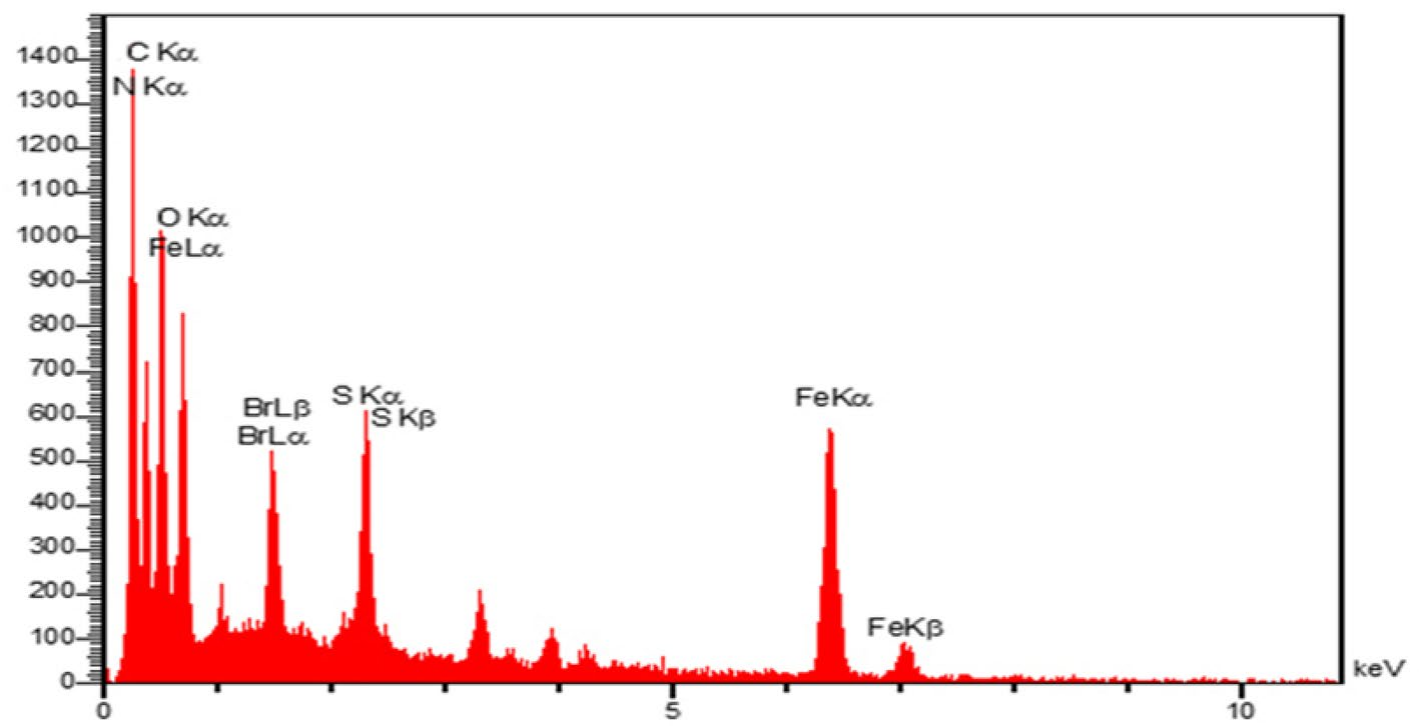
EDS spectra of recycled catalyst.

**Figure 17:**
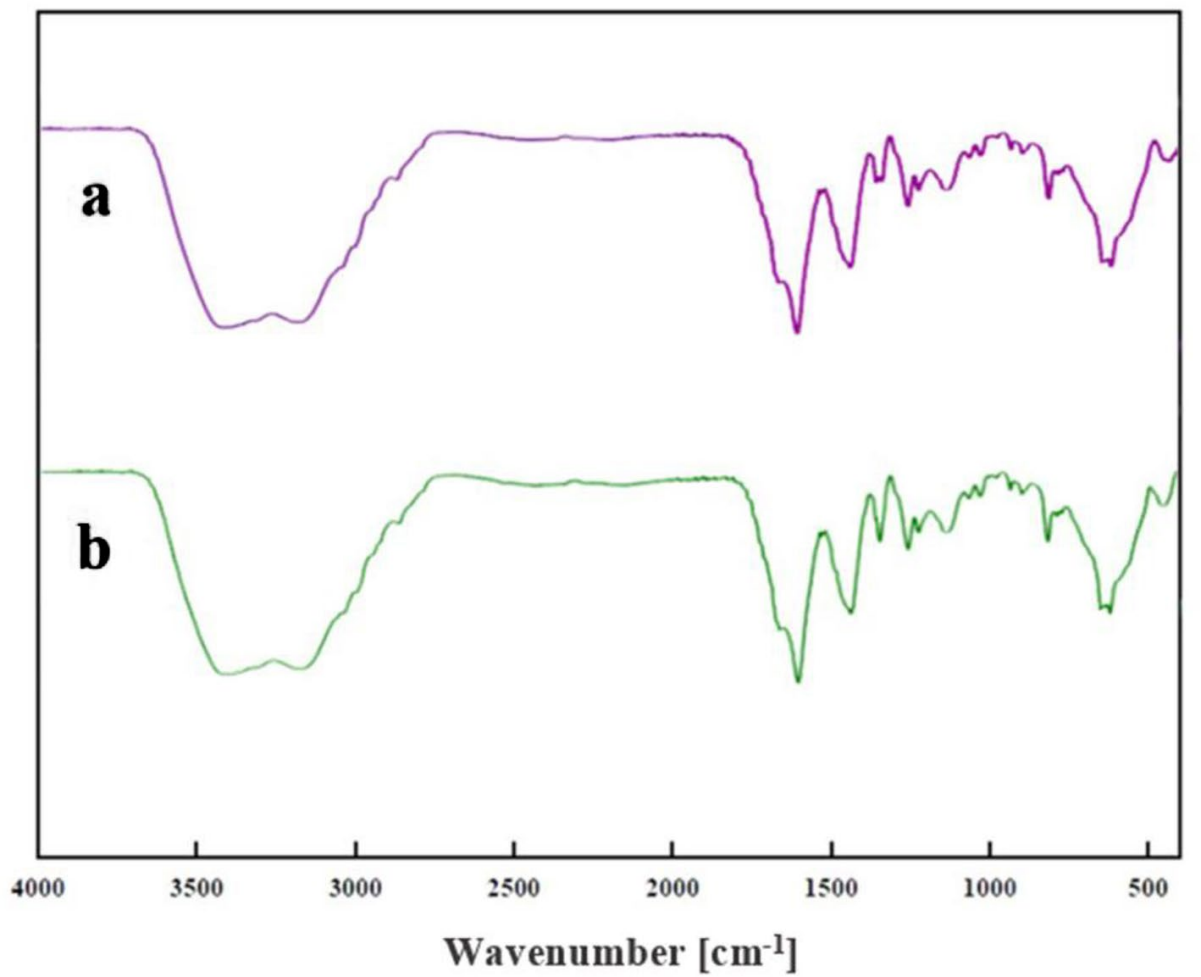
The FT-IR spectra of (**a**) catalyst, (**b**) recycled catalyst.

Based on the use of captopril on the catalyst surface and its acidic groups, it leads to spatial congestion with other molecules on the catalyst surface. This spatial congestion can result in the closure of the proximity space around the C–Br bond. It can hinder the interaction of the C–Br bond with other molecules in the reactants, thereby reducing its role. When the catalyst surface is filled with adsorbent groups, the free space for molecular interactions decreases. This can cause congestion and a reduction in interactions involving a specific bond in the reactant molecules. As a result, interactions that rely on that bond for catalytic activity decrease, leading to a diminished role of the C–Br bond in the reaction. Ultimately, the reactive components interact with the free acidic groups in the structure of captopril, promoting reaction progress and high product yield. However, if the reactive components were to react with C–Br instead, it would not result in high product yields. Refer to Tables 2 and 4 to see the yield of the products. In addition to this, the recovered catalyst (Fe_3_O_4_@g-C_3_N_4_-Pr-Cap) was characterized by Energy-dispersive X-ray spectroscopy (EDS) and FT-IR (Figs. 16, and 17). The EDS spectrum displays the presence of Br atoms without significant change in comparison with fresh catalyst (Fig. 3b), which is a confirmation that the C–Br bond remains unchanged. The FT-IR of the fresh and recycled catalyst, Fe_3_O_4_@g-C_3_N_4_-Pr-Cap, is given in (Fig. 17). In Fig. 17b, the presence of stretching and bending vibrations of the C−Br at 700 cm^−1^ and 1100 cm^−1^, respectively; approves that C–Br bond remains unchanged.

## Conclusion

A magnetically recoverable and green catalyst was developed by immobilizing a safe and sustainable ligand on magnetic graphene nitride. The chemical nature and properties of the catalyst were characterized by different analysis techniques. From the values and the importance of the one-pot multicomponent reaction for the synthesis of heterocycles, we discovered that our environmentally friendly and sustainable catalytic system was super active for the synthesis of a wide-scope of 2-amino-4*H*‐chromenes and 1,2,3,6- tetrahydropyrimidine under mild and green conditions. Moreover, the common problem in these reactions is the formation of unwanted byproducts, which were not formed in the presented synthesis method. The Fe center of the catalyst makes it attractive due to the low cost, availability, and low toxicity of this metal. Magnetic core led to an easy recyclability process; moreover, the catalyst exhibited excellent reusability in at least five reaction runs.\

### Ethical approval

This work does not contain any studies with human participants or animals performed by any of the authors.

### Supplementary Information

Supplementary Information.

## Data Availability

All data generated or analysed during this study are included in this published article [and its supplementary information files].
