# Supplementary material for: Captopril supported on magnetic graphene nitride, a sustainable and green catalyst for one-pot multicomponent synthesis of 2-amino-4H-chromene and 1,2,3,6-tetrahydropyrimidine
Source: Sci Rep. 2023 Nov 23;13:20562. doi: 10.1038/s41598-023-47794-2 (PMC10667485; doi:10.1038/s41598-023-47794-2)
Supplement: Supplementary file 1 — Supplementary Information. [file 41598_2023_47794_MOESM1_ESM.docx]

**Supporting Information**

**Captopril supported on magnetic** **graphene nitride, a sustainable and green catalyst for one-pot multicomponent synthesis of** **2-amino-4*H*-chromene and 1,2,3,6-tetrahydropyrimidine**

Fatemeh Rezaei, Heshmatollah Alinezhad,^*^ Behrooz Maleki**

Department of Organic Chemistry, Faculty of Chemistry, University of Mazandaran, Babolsar, Iran; (email: [heshmat@umz.ac.ir](mailto:heshmat@umz.ac.ir); Heshmatollah Alinezhad; [b.maleki@umz.ac.ir](mailto:b.maleki@umz.ac.ir); Behrooz Maleki)

**^1^H and ^13^C NMR of selected products**


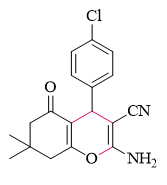


**4b**

**^1^H NMR, 400.13 MHz, DMSO:** δ_H_: 0.95 (3H, s, CH_3_), 1.03 (3H, s, CH_3_), 2.10 and 2.25 (2H, ABq, ^2^*J*_HH_= 16 Hz, CH_2_), 2.50 (2H, d, *^2^J*_HH_= 2 Hz, CH_2_), 4.19 (1H, s, CH), 7.06 (2H, s, NH_2_), 7.17 (2H, d, *^3^J*_HH_= 8.4 Hz, CH_Ar_), 7.35 (2H, d, *^3^J*_HH_= 8.4 Hz, CH_Ar_)


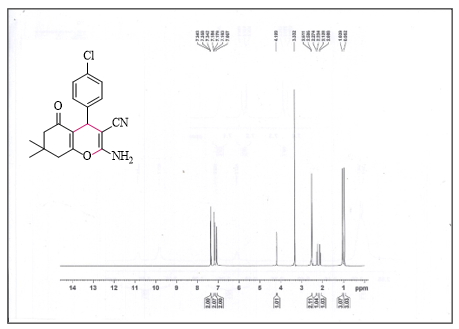


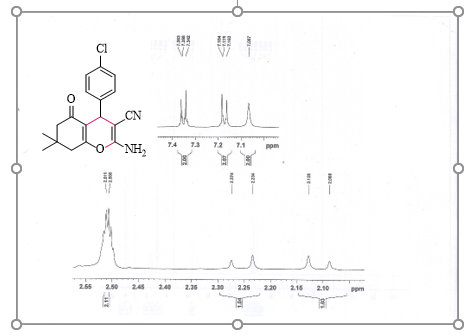


**Figure S4.** The ^1^H NMR spectrum of **4b**


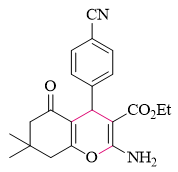


**4g**

**^1^H NMR, 400.13 MHz, DMSO:** δ_H_: 0.88 (3H, s, CH_3_), 1.03 (3H, s, CH_3_), 1.06 (3H, t, ^3^*J*_HH_= 6.8 Hz, CH_3_), 2.06 and 2.27 (2H, ABq, *^2^J*_HH_= 17.6 Hz, 16Hz, CH_2_), 2.48 and 2.56 (2H, ABq, *^2^J*_HH_= 18 Hz, 17.2Hz, CH_2_), 3.93 (2H, q, *^3^J*_HH_= 12 Hz, CH_2_), 4.57 (1H, s, CH), 7.33 (2H, d, *^3^J*_HH_= 8 Hz, CH_Ar_), 7.68 (2H, s, NH_2_), 7.68-7.70 (2H, m, CH_Ar_)

**^13^C NMR, 100.13 MHz, DMSO:** δ_C_: 14.6, 26.9, 29.0, 32.3, 34.4, 40.0, 50.2, 59.3, 77.0, 109.1, 114.8 (CN), 119.3, 129.3, 132.2, 152.4, 159.6, 163.1 (C=O), 168.1, 196.3 (C=O)


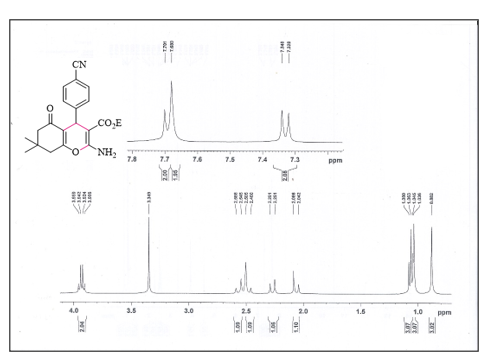


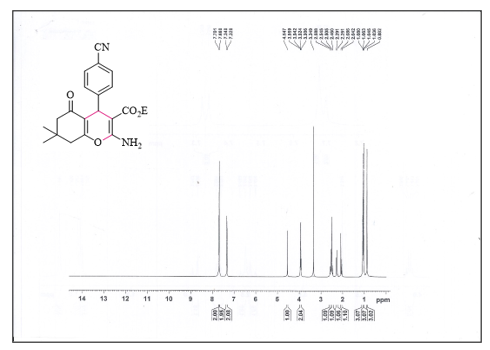


**Figure S5.** The ^1^H NMR spectrum of **4g**


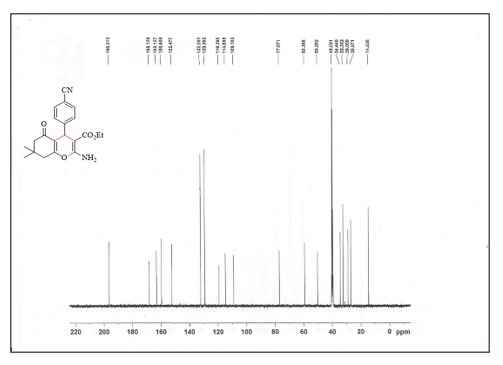


**Figure S6.** The ^13^C NMR spectrum of **4g**


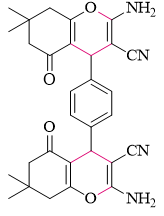


**4c**

**^1^H NMR, 400.13 MHz, DMSO:** δ_H_: 0.95 (3H, s, CH_3_), 0.98 (3H, s, CH_3_), 1.03 (6H, s, CH_3_), 2.14 and 2.24 (4H, ABq, *^2^J*_HH_= 16 Hz, CH_2_), 2.50 (4H, m, CH_2_), 4.14 (2H, s, CH), 6.96 (4H, s, NH_2_), 7.04 (4H, s, CH_Ar_)


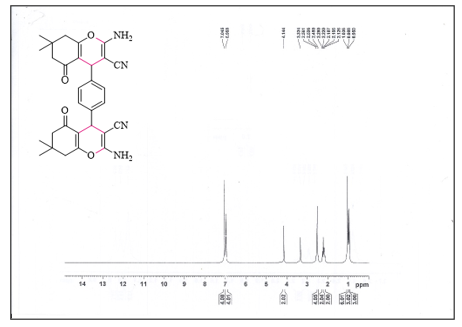


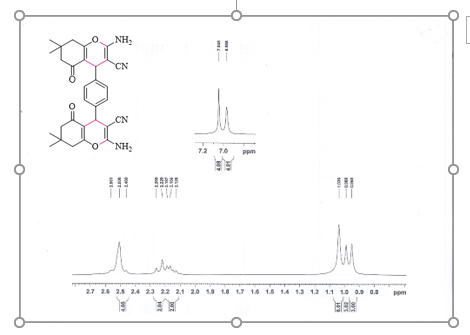


**Figure S7.** The ^1^H NMR spectrum of **4c**


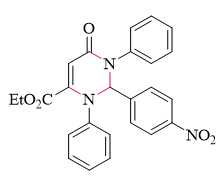


**7a**

**^1^H NMR, 400.13 MHz, CDCl_3_:** δ_H_: 1.03 (3H, t, *^3^J*_HH_= 7.2 Hz, CH_3_), 4.03 (2H, q, *^3^J*_HH_= 6.4 Hz, CH_2_), 5.94 (1H, s, CH), 7.11-7.15 (1H, m, CH_Ar_), 7.18-7.22 (3H, m, CH_Ar_), 7.26-7.30 (2H, m, CH_Ar_), 7.34-7.38 (2H, m, CH_Ar_), 7.44-7.48 (4H, m, CH_Ar_), 8.13 (2H, dt, *^3^J*_HH_= 4.8 Hz, *^4^J*_HH_= 2.4 Hz, CH_Ar_), 8.25 (1H, s, CH)

**^13^C NMR, 100.13 MHz, CDCl_3_:** δ_C_: 8.67, 55.28, 57.02, 102.65, 117.35, 117.92, 118.56, 120.03, 121.00, 123.35, 123.44, 123.94, 130.77, 132.96, 137.24, 139.52, 142.50, 158.59 (COO), 158.85 (CO)


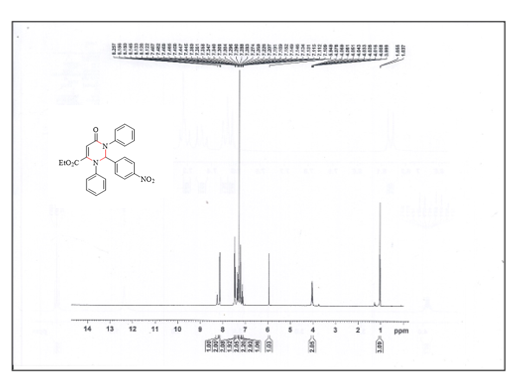


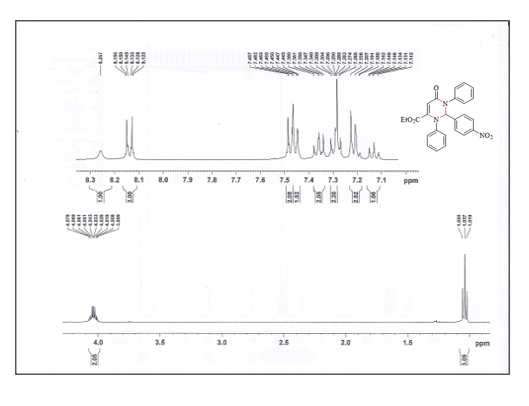


**Figure S8.** The ^1^H NMR spectrum of **7a**

**
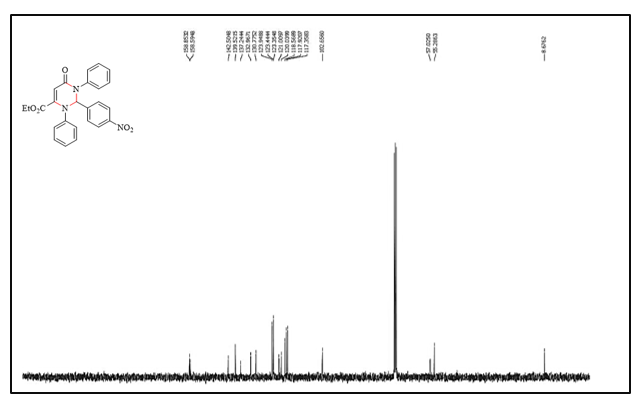
**

**Figure S9.** The ^13^C NMR spectrum of **7a**
